# CFD Application to Poultry Crate Design Improving Internal Airflow Velocities

**DOI:** 10.3390/ani15243633

**Published:** 2025-12-17

**Authors:** Victor Abreu de Lima, Jasson Fernandez Gurgel, Daniel Gurgel Pinheiro, Nítalo André Farias Machado, José Antonio Delfino Barbosa Filho, Antonio Velarde, Iran José Oliveira da Silva, Marcos Vinícius da Silva

**Affiliations:** 1 Department of Agricultural Engineering, Federal University of Ceará, Fortaleza 60450-760, CE, Brazil; victorabreudelima@gmail.com (V.A.d.L.); jassongurgel@alu.ufc.br (J.F.G.); 2 Department of Industry, Federal Institute of Education, Science, and Technology of Ceará, Fortaleza 60410-426, CE, Brazil; dgpinheiro@ifce.edu.br; 3 Chapadinha Science Center, Federal University of Maranhão, Chapadinha 65500-000, MA, Brazil; nitalo.farias@ufma.br (N.A.F.M.); mv.silva@ufma.br (M.V.d.S.); 4 IRTA, Animal Welfare, Veïnat de Sies, 17121, Monells, Catalonia, Spain; antonio.velarde@irta.cat; 5 Department of Biosystems Engineering, University of São Paulo (ESALQ/USP), Piracicaba 13418-900, SP, Brazil; iranoliveira@usp.br

**Keywords:** animal welfare, poultry, container, live transport, thermal stress

## Abstract

Poultry transport represents a significant animal welfare challenge, particularly when birds are exposed to heat stress during travel, a condition that can compromise physiological stability, performance, and survival. Despite the relevance of this issue, research on engineering improvements to poultry transport crates remains limited. In this study, four virtual models of poultry transport crates were evaluated to assess their potential to improve the thermal comfort internal airflow conditions. Computational Fluid Dynamics (CFD) simulations were conducted under three transport speeds, complemented by wind tunnel experiments using reduced-scale prototypes fabricated by additive manufacturing. The results demonstrated that the alternative crate 3 (AC3) model presented exhibited superior internal average airflow velocities (IAFV) across all speeds, including a 32.85% increase compared to the conventional crate at 60 km/h. Wind tunnel testing confirmed significant differences among crate designs. AC3 showed lower air temperature than AC1 and reduced relative humidity compared to CC and AC2. Thermal comfort indices supported these findings, with AC3 presenting the lowest THI and enthalpy, indicating a less stressful microclimate. In terms of airflow, AC2 and AC3 achieved higher IAFV (19.27 ± 8.49 m/s and 19.30 ± 4.80 m/s) than CC and AC1. AC3 also had the lowest dynamic pressure, suggesting reduced airflow resistance and more efficient aerodynamics. Therefore, improved crate geometry and increased ventilation surface can enhance airflow distribution, potentially reduce heat accumulation and improve animal welfare. However, further studies involving live birds, realistic stocking densities, and full-scale trailer simulations are required to validate these benefits under commercial transport conditions.

## Simple Summary

Poultry transport over long distances is a common practice and involves billions of birds each year. However, many birds suffer stress during travel, especially when the weather is hot and there is not enough fresh air inside the transport crates. Heat stress can harm the animals, affect their comfort, and reduce meat quality, which also causes economic losses for farmers and the industry. In this study, we combined computer simulations with wind tunnel experiments on reduced-scale prototypes to evaluate airflow in four crate designs. We compared the crate normally used in Brazil with three new designs at different truck speeds. One design, which had more open space on its sides, showed much better airflow in all situations. This means it may help keep birds cooler and more comfortable during transport. Although our results show promise, more research with live birds is needed to fully understand how these improvements can protect animals during real transport conditions.

## 1. Introduction

Worldwide, poultry transport poses a complex animal welfare challenge. The transport operation involves a significant number of birds, in the order of billions annually, and can occur several times throughout the production cycle [1,2,3,4]. During the transport operations, several stressors, such as uncomfortable environmental conditions, high load density and improper handling of the birds during crating have the potential to cause stress and production losses [5,6,7,8]. According to the European Food Safety Authority (EFSA) report, the main welfare consequences during this phase include prolonged hunger and thirst, motion stress, sensory overstimulation, restriction of movement, injuries, as well as thermal stress from cold or heat [9].

In tropical and subtropical regions, such as Brazil and many other major poultry-producing countries, transport occurs under particularly challenging climatic conditions characterized by high ambient temperatures (frequently exceeding 30 °C) and high relative humidity (often above 70–80%), especially during the hottest months of the year [7,10,11]. These environmental conditions significantly increase the risk of heat stress in poultry, a species already highly susceptible due to its high metabolic rate, rapid growth, and limited thermoregulatory capacity resulting from intensive genetic selection and the absence of functional sweat glands [12,13,14]. Under such hot and humid conditions, even short-distance transport can lead to a rapid rise in temperature and humidity inside the crates, compromising birds’ ability to maintain thermal balance and increasing the incidence of hyperthermia, mortality (dead-on-arrival—DOA), and meat quality defects such as pale, soft, and exudative (PSE) meat [8,11,15,16,17].

Poultry are transported in thermodynamically inefficient crates [9,18]. The crate openings complicate bird placement, the structure transmits vibration during transit [19], and the equipment design influences ventilation rates and the internal microclimate [20,21]. This scenario is particularly concerning given the heightened susceptibility of poultry to heat stress, stemming from their elevated metabolic rate, and compromised thermoregulatory capacity [22]. Some modifications to the transport vehicles have been suggested to improve the environmental profile inside the transport crates, obtaining apart from the welfare improvements, positive results in reducing the incidence of meat quality problems [23]. However, naturally ventilated poultry transport vehicles are not able to provide uniform ventilation to all birds, and the microclimate within the trailer and crates can vary significantly depending on their position in vehicle, ambient temperature and humidity and truck speed [2,24].

The inadequate ventilation in the crates increased temperature and humidity causing challenges to birds maintain its body heat balance leading to animal welfare problems and, depending on the internal microclimate of the crate, leading to poultry death [10,12,16]. Some researchers are questioning the crates design, efficiency, and aerodynamic profile to provide adequate internal ventilation to birds [9,20,21,25]. Pinheiro et al. [21] concluded that, during broiler chicken transport in tropical climates, inter-crate ventilation can be enhanced using spacers; however, intra-crate airflow remains unchanged. The authors recommend further research into novel crate designs. Therefore, most ventilation challenges stem from structural obstructions within the crates, necessitating a comprehensive redesign.

In this context, there is a lack of research dedicated to improving the crates design aiming to provide better animal welfare conditions and is an eminent demand for new models that aim to optimize the aerodynamic profile of the containers, thus allowing greater air circulation and reducing the heat stress of the birds. To meet this need, a viable experimental proposal would be to perform this optimization *in silico*, through numerical simulations using Computational Fluid Dynamics (CFD), an approach that has demonstrated satisfactory results in solving problems in animal production systems [26,27,28,29,30]. The feasibility of this approach is supported by cost reduction, improvement of computers, increased performance of processors and advances in computer simulation software, which allows us to hypothesize that the use of CFD represents a promising alternative for the evaluation and improvement of the containers used to transport birds, allowing a detailed analysis of the air flow and the optimization of the aerodynamic profile. To complement the simulations, wind tunnel tests were also conducted using reduced-scale models of the evaluated crates. This study aimed to assess the thermal comfort and internal ventilation patterns of four poultry transport crate models (one standard and three alternatives) using computational simulation techniques and wind tunnel tests with reduced-scale prototypes fabricated through rapid prototyping.

## 2. Materials and Methods

### 2.1. Conception, Characterization and Determination of Models

**Figure 1:**
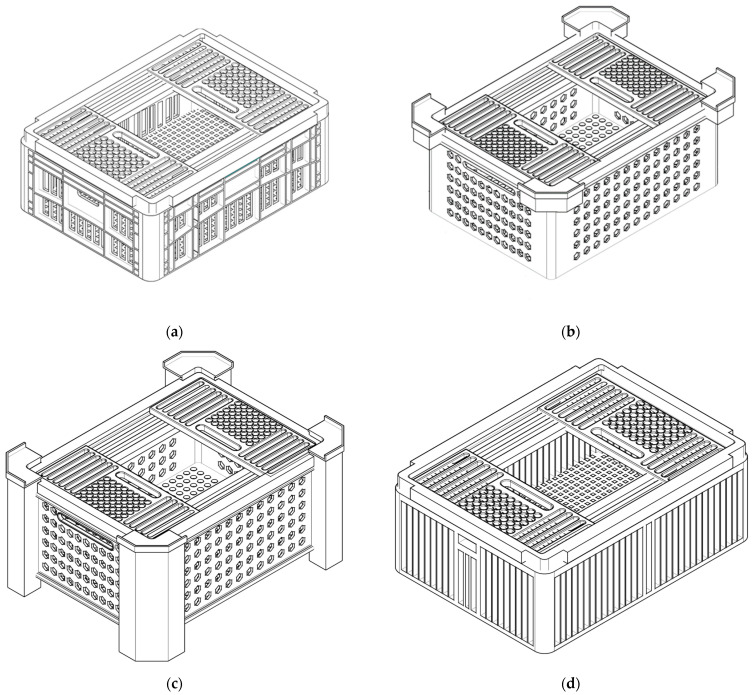
Virtual models of the poultry transport crates: (**a**) conventional crate (CC); (**b**) alternative crate 1 (AC1) with a rectangular base, vertical spacers on the top, and hexagonal side openings; (**c**) alternative crate 2 (AC2) with four integrated side pillars and hexagonal side openings; and (**d**) alternative crate 3 (AC3) with rectangular vertical side openings.

With the aim of developing thermodynamically more efficient poultry transport crates, a comprehensive state-of-the-art review was conducted, complemented by a systematic patent search across five databases (e.g., Espacenet, Patentscope, The Lens, Google Patents, and Brazil’s INPI), covering existing national and international solutions for live-animal transport. All relevant crate designs identified were presented and critically evaluated during an initial project meeting with the animal transportation research team. Subsequently, on-site visits were carried out under real commercial conditions in the metropolitan region of Fortaleza, Ceará, Brazil. These visits enabled direct consultation with poultry producers, professional transporters, and farm workers to identify the main practical limitations of the crates currently in use, particularly with regard to worker ergonomics and safety, operational efficiency, ease of manual and mechanical handling, and stacking stability. Considering the identified limitations, technical and practical feasibility, and mandatory compliance with Brazilian road-transport legislation [31], three virtual crate models (AC1, AC2, and AC3) were developed for *in silico* evaluation against the conventional crate (Figure 1), detailed individually below.

The virtual models were developed using AutoCAD^®^ software version 2023. The conventional crate (CC) was obtained from measuring the dimensions of a standard crate commonly used to transport poultry in Brazil, manufactured by Pisani Plásticos S.A. (Caxias do Sul, RS, Brazil), and then it was designed in the software. The other crates were developed based on the measurements of the Brazilian standard crate (Table 1) and had the patent registered in Industrial Property Nacional Institute (INPI) with number BR202021025473-0U2 and protocol number BR1020240187490. The design of the CC has a rectangular base and a row of horizontal openings at the top, a horizontal edge, and a row of horizontal openings at the bottom of the four sides of the box, including a horizontal opening, on the two smaller sides, to facilitate handling (Figure 1a). The first alternative model (AC1) was designed with a rectangular base, four vertical spacers positioned at the upper end’s sides with hexagonal openings different from the horizontal openings found in the CC (Figure 1b). The second alternative crate (AC2) was designed with a rectangular base and four pillars that extend from the top to the bottom, integrated with the crate, and hexagonal openings in the sides (Figure 1c). The same hexagonal opening found in the AC1 but with pillar-shaped spacers on the sides. The use of spacers and pillars integrated to the transport crates aims to facilitate handling during stacking and modify the ventilation pattern between crates as demonstrated in previous studies of computational analysis of load ventilation in poultry transport [21]. Finally, the third alternative crate (AC3) was based on the conventional crate; however, the horizontal edges were removed, resulting in larger and bigger vertical rectangular openings than the conventional design (Figure 1d). The hexagonal and square openings were chosen to compare between the conventional shapes and, according to computational fluid dynamics simulations for air collectors, the air flow rate is better in hexagonal collectors compared to square collectors [32].

### 2.2. Computational Domain, Grids and Numerical Simulation

CFD simulations were performed using ANSYS software version R1 2023. The computational domain used was 40.68 × 20.45 × 40.32 m (length × width × height). The crates models were sectioned and positioned in the center of the computational domain, and the airflow was directed towards the -Z vector. Unstructured meshes (tetrahedral and prismatic cells) were used in the computational domain. The broad dimension of the computational domain and the central position of each of the crates was chosen to improve the quality of analysis and facilitate the understanding of the air flow. Due to the characteristics of the study and to obtain accurate results, viscous models were used in the steady state, the turbulence modelling was done by RANS (Reynolds Averaging Navier–Stokes), and to deal with the complex geometry, which presented sharp curves and points and were aerodynamically little known, the k-ɷ SST model was chosen, recognized for its high qualities in these situations [27,29]. The walls of the crates were considered adiabatic, with zero heat flux and standard wall functions were used to save computational resources and enable the analysis. In the solution model, a Coupled-type scheme was adopted, to obtain more accurate results, together with high quality spatial discretization. To assess the accuracy of the results, error criteria were established and a maximum error of 10^−4^% was accepted for the energy value and 0.1% for the velocity and density components.

Transport crates are generally designed to hold between six and ten broilers, with seven birds per crate representing the most common stocking density [9]. In the present study, however, the CFD simulations were conducted using empty crates. The input speeds were defined based on the real and usual speeds of a poultry transport truck. Three velocities were selected, 8.33, 16.66 and 25 m/s (equivalent to 30, 60 and 90 km/h). For the simulations, boundary conditions were defined with an absolute temperature of 26.85 °C, relative humidity of 60%, and atmospheric pressure of 1 atm. The Reynolds number (Re) was determined using Equation (1). The k-ɷ SST, as defined, was modelled according to Equations (2) and (3) and its limiter formulation of turbulent viscosity.

(1) Re=Vlv 

where V is the velocity of the fluid (m/s), l is the characteristic length (m) and v is the kinematic viscosity (m^2^/s).

(2) $$\frac{\partial }{\partial t}(\rho k)+\frac{\partial }{\partial x_{i}}(\rho ku_{i})=\frac{\partial }{\partial x_{j}}(\Gamma_{k}\frac{\partial k}{\partial x_{j}})+G_{k}-Y_{k}+S_{k}\;+G_{b}$$

where ρ is the flow density, k is the turbulence kinetic energy, u is the flow velocity vector, $\Gamma_{k}$ represents the effective diffusivity of k, G is a production term, Y is the destruction term and S is a user-defined source term.

(3) $$\frac{\partial }{\partial t}(\rho \omega )+\frac{\partial }{\partial x_{i}}(\rho \omega u_{i})=\frac{\partial }{\partial x_{j}}(\Gamma_{\omega }\frac{\partial \omega }{\partial x_{j}})+G_{\omega }-Y_{\omega }+S_{\omega }\;+G_{\omega b\;\;}$$

where ω  is the specific dissipation rate.

For the treatment near to the walls, a wall function model was adopted and to calculate the wall dimensionless distance, Equation (4) was used and the friction velocity was defined by Equation (5):

(4) $$y^{+}\;=\;\rho \;\times \;\mathrm{ut}\;\times \;\frac{y}{\mu }$$

where $y^{+}$ is the wall dimensionless distance, ut is the friction velocity, y is the wall distance and μ is the molecular viscosity.

(5) $$\mathrm{ut}={\left(\frac{\tau_{w}}{\rho }\right)}^{1/2}$$

Finally, the modelling, admitting the mesh, was defined according to Equations (6)–(8):

(6) $$U^{*}\;=\;\frac{1}{k}\ln(Ey^{*})$$

(7) $$U^{*}=\frac{U_{p}C_{\mu }^{1/4}k_{p}^{1/2}}{\tau_{w}/\rho }$$

(8) $$y^{*}=\frac{\rho C_{\mu }^{1/4}k_{p}^{1/2}y_{p}}{\mu }\;$$

which: k is the Von Karman constant, E is an empirical constant, $U_{p}$ is the average fluid velocity at the centroid of the adjacent cell wall and the variables with index p refer to the centroid of the adjacent cell in the wall.

### 2.3. Wind Tunnel Tests

**Figure 2:**
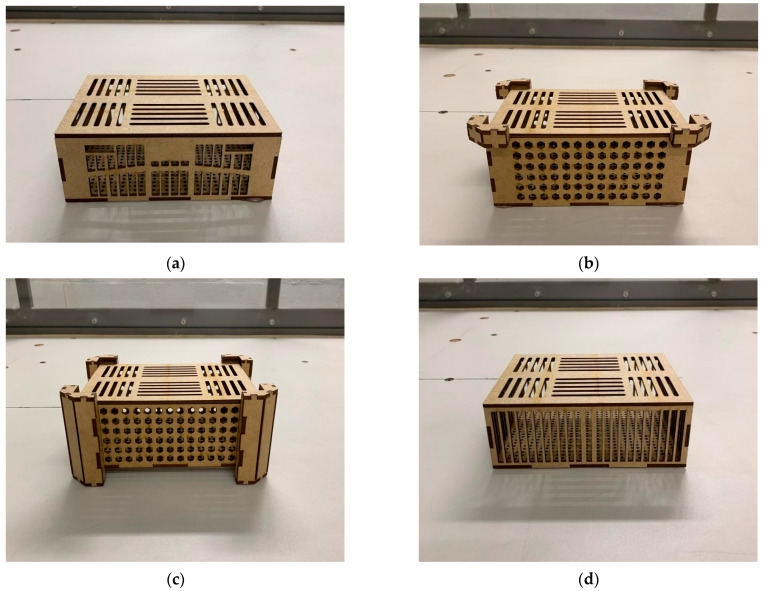
Prototypes of poultry transport crate models: (**a**) conventional crate (CC); (**b**) alternative crate 1 (AC1) with a rectangular base, vertical spacers on the top, and hexagonal side openings; (**c**) alternative crate 2 (AC2) with four integrated side pillars and hexagonal side openings; and (**d**) alternative crate 3 (AC3) with rectangular vertical side openings.

The virtual crate models were manufactured using computer numerical control (CNC) laser cutting equipment (TAICO Laser knowledge 1060, TAICO Technology, Shenzhen, China), with 3 mm medium-density fiberboard (MDF) selected for its quality, ease of handling, and low cost, enabling rapid prototyping at a 1:5 scale (Figure 2). Each crate required six MDF panels (one for each side, the top, and the bottom, plus additional interlocking pieces). After cutting, the pieces were fitted or glued together, and the spaces between the joints were filled with modeling clay to prevent unwanted air circulation.

After fabrication, the physical crate models were tested in the wind tunnel of the Aerodynamic Testing Laboratory (LEA) at the State University of Ceará (UECE). The UECE wind tunnel covers an area of more than 500 m^2^, with a test section of 1 m^2^, capable of generating airflow velocities up to 150 km/h while maintaining turbulence intensity below 2%. All four poultry transport crate models were evaluated. Each wind tunnel test lasted ten minutes per crates. Measurements were taken from the front and side views at the same flow velocities used in the CFD simulations. Prior to testing, local temperature and pressure were measured, and the pressure sensor was calibrated. The crates were then positioned and secured in the tunnel, followed by the placement of a Pitot tube for pressure readings. A single Pitot tube was used due to the crate dimensions and the limited space available for equipment positioning. The tube was inserted into each crate to a depth of 31.4 mm. Subsequently, additional sensors were installed to measure the microclimatic parameters described below. Once all adjustments were completed, the wind tunnel was activated, and data were collected on temperature, relative humidity, and internal average air flow velocity, and dynamic pressure. The microclimate was characterized using the Temperature–Humidity Index (THI), calculated according to Equation (9) proposed by Berman et al. [33]. Furthermore, the Enthalpy Index (H, kJ/kg dry air) was calculated according to the equation proposed by Rodrigues et al. [34], from Equation (10).

(9) THI=3.43+(1.058×AT)−(0.293×RH)+(0.0164×AT×RH)+35.7

(10) $$H=1.006\times \mathrm{AT}+\frac{\mathrm{RH}}{\mathrm{PB}}\times 10^{\left(7.5\times \mathrm{TA}/237.3+\mathrm{TA}\right)}\times (71.28+0.052\times \mathrm{AT})$$

where AT is the air temperature (°C), RH is the relative humidity (%), and PB is the local barometric pressure (mmHg), considered as 1 atm.

### 2.4. Statistical Analysis

The assumptions of normality and homogeneity of variances were tested using the Shapiro–Wilk test. When both assumptions were met (air temperature, relative humidity, THI, Enthalpy Index, dynamic pressure), differences among the four crate designs were analyzed using one-way ANOVA followed by Tukey’s multiple comparisons test. When assumptions were violated (internal average airflow velocities), the non-parametric Kruskal–Wallis’s test followed by Dunn’s post hoc test was applied. Outliers in wind tunnel tests were identified and discarded using the Robust Regression and Outlier Removal method, with an aggressiveness criterion of 1%. All statistical analyses were performed using Statistical Analysis Software (SAS) OnDemand for Academics Version 9.4, adopting *p* < 0.05 as the threshold for statistical significance.

## 3. Results

The AC3 crate had more surface area of openings, with 254.7% and 297.8% more open areas on the larger side than the CC and the AC1 and AC2, respectively. On the smaller side, the AC3 presented 260.9% and 223.9% more open areas than the CC and the AC1 and AC2, respectively (Table 2).

**Figure 3:**
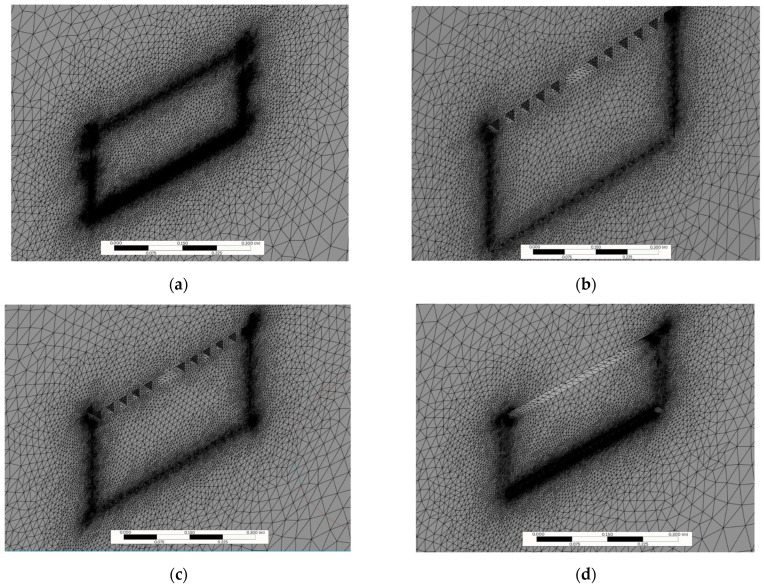
Poultry transport crate meshes: (**a**) conventional crate (CC); (**b**) alternative crate 1 (AC1) (**c**) alternative crate 2 (AC2); and (**d**) alternative crate 3 (AC3).

The meshes used for the simulations presented an average smoothing of 0.85476, that is, very close to 1 and the orthogonal quality presented an average of 0.79781, indicating that the simulated elements have a satisfactory geometry (Figure 3). The Reynolds numbers obtained for simulated velocities of 8.33, 16.66 and 25 m/s were 1,210,898, 807,265 and 403,632, respectively.

The internal average of airflow velocities across the crates during the simulation are presented in Table 3. The results showed that the CC presented higher internal average of airflow velocities compared to the alternative crates AC1 and AC2 in all three simulation speeds. On the other hand, the alternative model AC3 showed, at all simulation speeds, higher values of internal average of airflow velocities compared to the CC. Notably, at the speed of 60 km/h, which represents the average vehicle speed when transporting live poultry, the AC3 demonstrated a 32.85% increase in internal average of airflow velocities compared to the CC.

**Figure 4:**
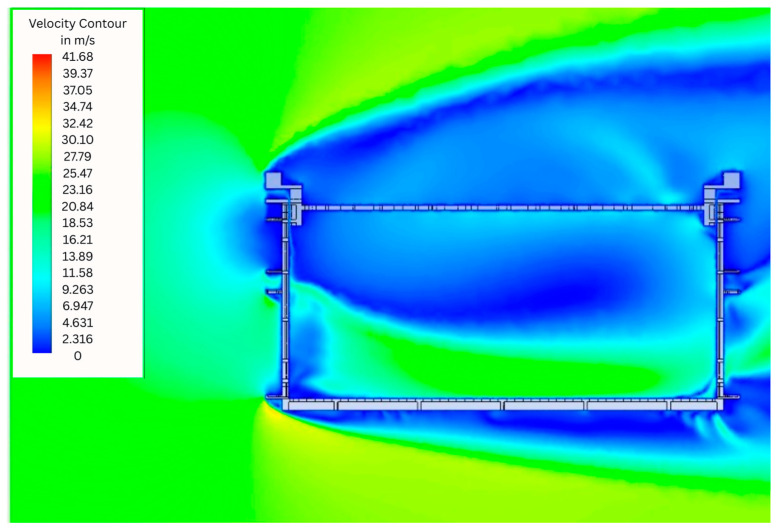
Simulation results showing the air flow profile with colours indicating the air velocity in m/s in the Conventional Crate (CC).

Figure 4 shows simulation results showing the air flow profile with colors indicating the air velocity in m/s in the Conventional Crate (CC). The results reveal high-velocity external flow along the leading edge, with significant airflow separation and recirculation zones downstream of the crate. On the other hand, internally, stagnant regions (blue) and air flow velocity equal to zero dominate, mainly in the upper part of the air inlet, in the center and in the upper part of the air outlet of the crate. These stagnation points indicated areas of lower air circulation. Therefore, it is possible to observe that most of the moving air was concentrated and moved mainly near the base of the crate.

**Figure 5:**
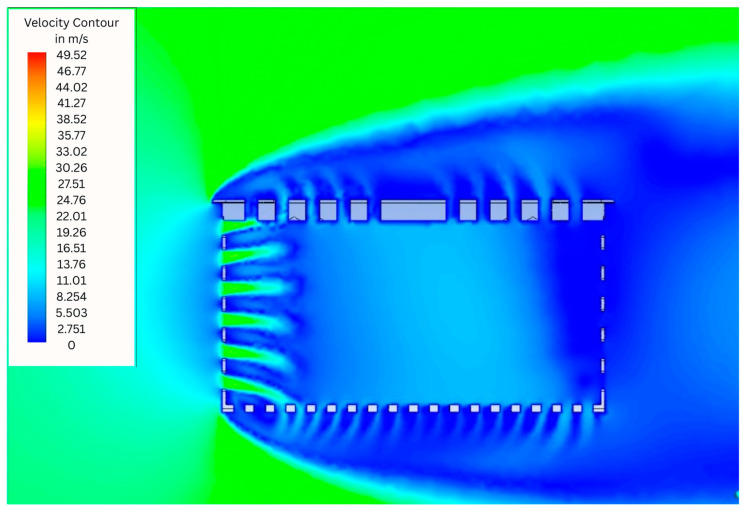
Simulation results showing the air flow profile with colours indicating the air velocity in m/s in the Alternative Crate 1 (AC1).

**Figure 6:**
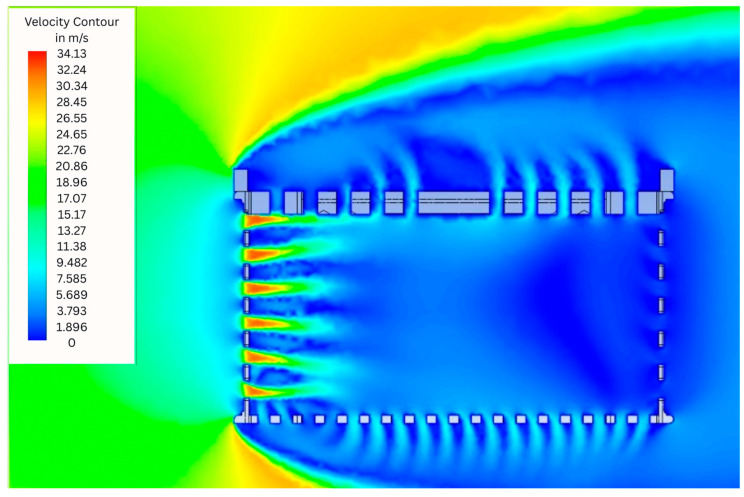
Simulation results showing the air flow profile with colours indicating the air velocity in m/s in the Alternative Crate 2 (AC2).

Examination of AC1 (Figure 5) and AC2 (Figure 6), the simulated profile presented a wider air distribution at the inlet compared to the CC. This wider inlet flow suggests improved initial air capture and a more favorable entry geometry. However, despite this improvement, significant obstructions persist in the initial region, severely restricting airflow penetration into the crate. Internally, air velocity remains near zero across most of the volume, as indicated by the predominant light and dark blue regions in the velocity contour plots. This near-stagnant condition extends throughout the central and upper zones, with minimal airflow reaching the interior or exiting the crate. Consequently, while AC1 and AC2 achieve better inlet air spreading than the CC, the internal ventilation remains critically limited, failing to promote effective air renewal within the crate.

**Figure 7:**
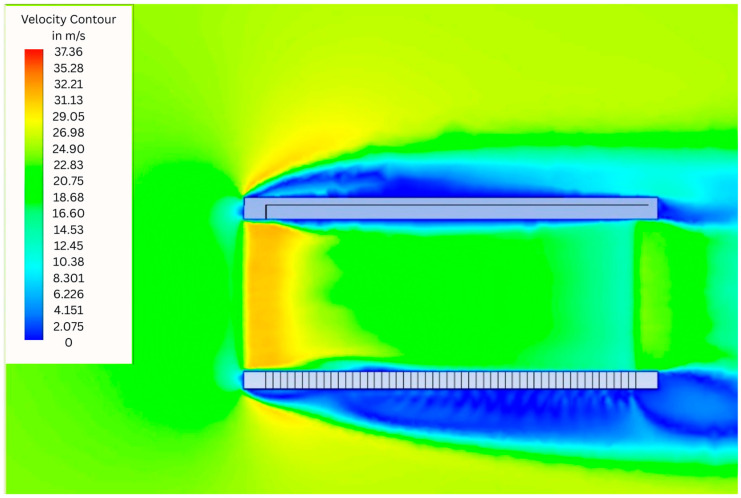
Simulation results showing the air flow profile with colours indicating the air velocity in m/s in the Alternative Crate 3 (AC3).

In the AC3 (Figure 7) it was evident that the shape of the sides of this model allows a fluid and unobstructed air intake. This design feature enables fluid entry with minimal resistance, promoting uniform and efficient internal airflow distribution. As a result, internal air velocities are significantly higher than in the CC, AC1, and AC2 models, with extensive regions of moderate to high flow clearly visible in the green, yellow, and red zones of the velocity contour plot. These areas indicate active air circulation throughout the crate volume, including the central and upper regions, in stark contrast to the near-stagnant conditions observed in the other designs.

Statistical analysis revealed significant differences among crate designs when compared to the conventional crate (CC) during wind tunnel testing (Table 4). Air temperature was significantly lower in AC3 compared to AC1. Regarding relative humidity, no significant difference was observed between AC2 and CC; however, both crates exhibited significantly higher RH values than AC3. THI values did not differ among CC, AC1, and AC2. In contrast, AC3 presented a significantly lower THI value, indicating a potentially less thermally stressful microclimate. A similar trend was observed for the enthalpy index, with AC1 and AC2 showing the highest values, followed by CC and, lastly, AC3, which exhibited the lowest enthalpy. In terms of internal airflow, AC2 and AC3 demonstrated superior ventilation performance, with internal average air flow velocities (IAFV) of 19.27 ± 8.49 m/s and 19.30 ± 4.80 m/s, respectively. These values were significantly higher than those recorded for CC and AC1, highlighting the enhanced airflow distribution in these alternative designs. Additionally, dynamic pressure (DP) values revealed important aerodynamic differences among the crate models. AC3 exhibited the lowest mean dynamic pressure, compared to higher values in CC, AC1, and AC2. This suggests that AC3 offers less resistance to airflow, facilitating smoother and more efficient air passage through the crate.

## 4. Discussion

This study investigated the internal ventilation patterns of four poultry transport crate models, combining CFD simulations with wind tunnel experiments. To the best of our knowledge, this is the first study to investigate airflow dynamics inside poultry transport crates by combining experimental and numerical approaches, in which new crate prototypes were designed, built, and evaluated with the primary goal of optimizing crate aerodynamics to improve the thermal and ventilation conditions experienced by birds during transport. By simulating crate aerodynamics at different travel speeds, the results consistently demonstrated that the conventional Brazilian crate (CC) exhibited reduced opening surface area, leading to non-uniform airflow and zones of low velocity, particularly in the upper region where birds’ heads are positioned.

For broilers, the recommended comfort zone is typically 21–27 °C with relative humidity between 50 and 70%, conditions that support effective thermoregulation and reduce mortality risk [9,35]. However, transport frequently occurs outside this range. Under such circumstances, adequate air renewal becomes essential, as insufficient ventilation leads to localized moisture buildup and the formation of thermal cores, which are strongly associated with increased mortality during transport [7,11,36]. With this in mind, the airflow restrictions observed in conventional crates can reduce air exchange and compromise environmental quality by promoting saturation (i.e., the accumulation of relative humidity), a condition that impairs the birds’ thermoregulation [11,15]. Moreover, these restrictions increase the concentration of gases (e.g., ammonia, methane, and carbon dioxide), as well as dust and particulate matter [37].

Prior attempts to enhance ventilation have focused on inter-crate airflow. Pinheiro et al. [21] assessed spacer use between transport crates through CFD simulations and wind tunnel experiments. The prototype, comprising four support columns, two 230 mm bars, two 630 mm bars, and four column-support bungs, was designed to create horizontal and vertical airflow corridors. Although this configuration improved circulation between crates, it did not significantly enhance internal ventilation around the birds. In a complementary in silico study, Pinheiro et al. [25] demonstrated that crate stacking configurations directly influence trailer ventilation. The authors showed that arrangements with a central aisle promote greater inter-crate airflow compared to conventional stacking. However, this setup reduces load capacity by approximately 20% and, similarly, fails to substantially improve internal airflow within occupied crates.

In their study, Vinco et al. [20] compared the transport of broilers in conventional crates and in crates with double the height, observing in vivo that the increased internal space led to greater stress, agitation, and post-mortem injuries such as bruises and scratches. Contrary to the initial expectation of improved welfare, the modified crates did not provide physiological or behavioral benefits and, in several cases, intensified stress and injury. Critically, this scenario supports the hypothesis that poultry are being transported in thermodynamically inefficient crates. In poultry housing, Ma et al. [38] demonstrated that symmetrical redistribution of exhaust fans enhanced thermal uniformity by up to 88.3%. Emesu et al. [37] showed that natural ventilation in broiler barns is strongly influenced by building orientation and roof geometry, with arched and gabled structures outperforming cantilevered designs. These studies converge on a fundamental aerodynamic principle: airflow efficiency is governed by geometry, orientation, and boundary conditions. We extend this paradigm to live poultry transport crates, aiming to develop designs that promote uniform airflow throughout the entire internal area.

When analyzing the simulated profile of the AC3, it is evident that the opening geometry of the sides of this model, with larger and more open vertical rectangular openings, provides a fluid and unobstructed air intake compared to the CC. CFD simulations showed that, the proposed AC3 model stands out, as it presented has on aver-age, 2.6 times more surface area of openings when compared to the other crates tested and better internal air flow profile during simulated transport conditions, when com-pared to the CC and the other proposed models, AC1 and AC2. Wind tunnel experiments validated the CFD outcomes and revealed performance differences among crate designs. Statistical analyses showed that AC3 maintained significantly lower relative humidity than the conventional crate. Thermal indices reinforced these results: AC3 exhibited the lowest THI and enthalpy values, indicating a less thermally stressful microclimate. In terms of airflow, AC2 and AC3 achieved significantly higher internal average airflow velocities (IAFV ≈ 19 m/s) than CC and AC1, underscoring their improved ventilation efficiency. Notably, AC3 also presented the lowest dynamic pressure, suggesting reduced airflow resistance and smoother aerodynamic behavior. Together, these findings highlight crate geometry as a promising avenue for further research aimed at improving bird transport systems.

These results are in line with the findings of Farouk [32], who used computational fluid dynamics to investigate air collectors installed on the roof of buildings. This study concluded that air collectors with square openings have higher mean internal air velocity. In the case of transport crates, the AC3 model, with a profile of bigger square openings, demonstrated greater efficiency in internal ventilation, proving to be more promising to improve the thermal comfort of the birds. In the study conducted by Gilkeson et al. [26], who analyzed the transport of sheep, it was concluded that the movement of the vehicle during transport is sufficient to promote air circulation inside the compartments, and this factor could be used to reduce the impact of thermal stress on birds during transport. However, the results of the present study demonstrate that crate design directly governs internal airflow during poultry transport, with air circulation depending not only on vehicle speed but also on the aerodynamic performance determined by the geometry and dimensions of the crate openings.

Although the velocities measured in AC3 prototypes were much higher, these values reflect reduced-scale wind tunnel conditions and should not be interpreted as direct equivalents to commercial transport environments. Another critical factor is the presence of birds and stocking density, which drastically alter internal microclimates. Studies have shown that live animals increase heat and moisture production, modify airflow distribution, and elevate gas concentrations [39,40]. Our simulations and wind tunnel tests were conducted with empty crates and, therefore, cannot fully capture these biological interactions. Moreover, commercial transport involves stacked crates within trailers, where recirculation zones, stacking effects, and vehicle aerodynamics further influence ventilation [26]. Research on swine and cattle transport has demonstrated that trailer geometry, airflow devices, and vehicle orientation significantly affect internal microclimates [18,41,42,43]. These findings highlight the complexity of scaling crate-level improvements to full transport systems. Future studies must incorporate live birds or realistic porous-zone representations, as well as full-scale trailer simulations and in situ measurements, to validate welfare benefits under commercial conditions.

## 5. Conclusions

This study highlights the pivotal role of crate design in shaping internal airflow dynamics during poultry transport. Using CFD simulations and wind tunnel experiments on reduced-scale prototypes, we showed that the conventional Brazilian crate exhibits limited ventilation capacity and uneven airflow distribution, particularly in regions critical to thermoregulation. In contrast, the alternative AC3 mode, with a larger surface area of lateral openings, consistently improved average internal airflow velocity across all simulated transport speeds. While the results offer valuable aerodynamic insights at the crate scale, further investigations incorporating live-bird physiology, dynamic thermal loads, and full-scale transport scenarios are essential to validate welfare outcomes and different airflow patterns.

## Figures and Tables

**Table 1:** Dimensions in the conventional crate (CC), alternative crate 1 (AC1), alternative crate 2 (AC2), and alternative crate 3 (AC3).

Item	External Dimensions (Length × Width × Height in cm)	Internal Dimensions (Length × Width × Height in cm)
CC	77 × 57 × 32	71.5 × 54 × 24
AC1	79 × 59 × 31	74 × 54 × 25
AC2	79 × 59 × 31	74 × 54 × 25
AC3	77 × 57 × 32	71.5 × 54 × 24

**Table 2:** Areas in the conventional crate (CC), alternative crate 1 (AC1), alternative crate 2 (AC2), and alternative crate 3 (AC3).

Crate Design	TSALS (cm^2^)	TSAOLS (cm^2^)	TSASS (cm^2^)	TSAOSS (cm^2^)
CC	4389	533.1	1824	345.8
AC1	4661	456	1829	403
AC2	4661	456	1829	403
AC3	4389	1358	1824	902.5

TSALS: Total surface area of larger side; TSAOLS: Total surface area of openings in larger side; TSASS: Total surface area of smaller side; TSAOSS: Total surface area of openings in smaller side.

**Table 3:** Internal average airflow velocity (m/s; mean ± SEM) inside the conventional crate (CC), alternative crate 1 (AC1), alternative crate 2 (AC2), and alternative crate 3 (AC3) at different simulated speeds obtained by computational fluid dynamics.

Crate Design	30 km/h	60 km/h	90 km/h
CC	3.2 ± 0.11 ^b^	7.58 ± 0.23 ^b^	11.37 ± 0.34 ^b^
AC1	1.83 ± 0.05 ^c^	3.51 ± 0.11 ^c^	6.43 ± 0.19 ^c^
AC2	1.69 ± 0.07 ^c^	3.64 ± 0.14 ^c^	5.09 ± 0.15 ^c^
AC3	4.99 ± 0.15 ^a^	10.07 ± 0.30 ^a^	14.92 ± 0.45 ^a^

Different superscript letters within a column indicate significant differences by test Kruskal–Wallis followed by Dunn’s test, *p* < 0.05.

**Table 4:** Air temperature (TA), relative humidity (RH), temperature-humidity index (THI), enthalpy index (H), internal average air flow velocity (IAFV), and Dynamic pressure (DP) recorded the conventional crate (CC), alternative crate 1 (AC1), alternative crate 2 (AC2), and alternative crate 3 (AC3) during wind tunnel tests.

Crate Design	TA (°C)	RH (%)	THI	H (kJ/kg)	IAFV (m/s)	DP (Pa)
CC	35.73 ± 0.21 ^ab^	36.88 ± 0.34 ^a^	87.03 ± 0.25 ^a^	69.95 ± 0.32 ^b^	16.14 ± 7.70 ^b^	226.17 ± 75.71 ^b^
AC1	36.02 ± 0.24 ^a^	36.42 ± 0.36 ^ab^	87.37 ± 0.34 ^a^	70.35 ± 0.56 ^a^	17.02 ± 7.94 ^b^	244.33 ± 79.99 ^a^
AC2	35.90 ± 0.20 ^ab^	37.00 ± 0.43 ^a^	87.34 ± 0.29 ^a^	70.54 ± 0.51 ^a^	19.27 ± 8.49 ^a^	246.33 ± 79.32 ^a^
AC3	35.35 ± 0.20 ^b^	35.95 ± 0.31 ^b^	86.13± 0.25 ^b^	68.01 ± 0.32 ^c^	19.30 ± 4.80 ^a^	156.83 ± 51.79 ^c^

Values are expressed as mean ± standard error of the mean. Different superscript letters within a column indicate statistically significant differences (*p* < 0.05). One-way ANOVA followed by Tukey’s multiple comparisons test was applied to all variables, except internal average air flow velocity (IAFV), which was analyzed using the Kruskal–Wallis’s test followed by Dunn’s post hoc test.

## Data Availability

The data used in this research is confidential, as it is a funded study that supports other research projects. However, the authors can make the data available upon request.

## References

[1] Sangoremi A.A., Olayanju O.K., Koko D.T., Bamigboye O., Alfred M.O. (2025). Management of Poultry and Poultry Wastewater. Strategic Management of Wastewater from Intensive Rural Industries.

[2] Weeks C.A., Tuyttens F.A.M., Grandin T. (2019). Poultry Handling and Transport. Livestock Handling and Transport.

[3] Benincasa N.C., Sakamoto K.S., Da Silva I.J.O., Lobos C.M.V. (2020). Animal Welfare: Impacts of Pre-Slaughter Operations on the Current Poultry Industry. J. Anim. Behav. Biometeorol..

[4] Allen V.M., Burton C.H., Wilkinson D.J., Whyte R.T., Harris J.A., Howell M., Tinker D.B. (2008). Evaluation of the Performance of Different Cleaning Treatments in Reducing Microbial Contamination of Poultry Transport Crates. Br. Poult. Sci..

[5] Wurtz K.E., Herskin M.S., Riber A.B. (2024). Water Deprivation in Poultry in Connection with Transport to Slaughter—A Review. Poult. Sci..

[6] Adam S.Y., Ahmed A.A., Jammaa M.H., AL Makhmari M.R., Husien H.M., Essa M.O.A., Elwan H., Shehab-El-Deen M., Elnesr S.S., Saleh A.A. (2025). Traditional Transportation Methods and Their Influence on Local Chicken Welfare, Behavior, and Blood Profiles: A Policy Considerations. Vet. Sci..

[7] Dos Santos V.M., Dallago B.S.L., Racanicci A.M.C., Santana P., Bernal F.E.M. (2017). Effects of Season and Distance during Transport on Broiler Chicken Meat. Poult. Sci..

[8] Wang P., Zhao Y., Jiang N., Li K., Xing T., Chen L., Wang X., Tang Y., Xu X. (2016). Effects of Water-Misting Spray Combined with Forced Ventilation on Heat Induced Meat Gelation in Broiler after Summer Transport. Poult. Sci..

[9] Nielsen S.S., Alvarez J., Bicout D.J., Calistri P., Canali E., Drewe J.A., Garin-Bastuji B., Gonzales Rojas J.L., Gortázar Schmidt C., Herskin M. (2022). Welfare of Domestic Birds and Rabbits Transported in Containers. EFSA J..

[10] Abidin Z.Z., Sulaiman N.F.A., Ramiah S.K., Awad E.A., Idrus Z. (2022). The Effect of Water Shower Spray on Stress Physiology and Mortality in Broiler Chickens Subjected to Road Transportation under the Hot and Humid Tropical Condition. Trop. Anim. Health Prod..

[11] dos Santos V.M., Dallago B.S.L., Racanicci A.M.C., Santana Â.P., Cue R.I., Bernal F.E.M. (2020). Effect of Transportation Distances, Seasons and Crate Microclimate on Broiler Chicken Production Losses. PLoS ONE.

[12] Miftakhutdinov A.V., Sayfulmulukov E.R., Ponomareva T.A. (2022). Heat and Transport Stress in Industrial Poultry: Problems and Solution. Russ. Agric. Sci..

[13] Di Martino G., Capello K., Russo E., Mazzucato M., Mulatti P., Ferrè N., Garbo A., Brichese M., Marangon S., Bonfanti L. (2017). Factors Associated with Pre-Slaughter Mortality in Turkeys and End of Lay Hens. Animal.

[14] Jacobs L., Delezie E., Duchateau L., Goethals K., Ampe B., Lambrecht E., Gellynck X., Tuyttens F.A.M. (2016). Effect of Post-Hatch Transportation Duration and Parental Age on Broiler Chicken Quality, Welfare, and Productivity. Poult. Sci..

[15] Rui B.R., de Angrimani D.S.R., Silva M.A.A. (2011). da Pontos Críticos No Manejo Pré-Abate de Frango de Corte: Jejum, Captura, Carregamento, Transporte e Tempo de Espera No Abatedouro. Ciênc. Rural..

[16] Schwartzkopf-Genswein K.S., Faucitano L., Dadgar S., Shand P., González L.A., Crowe T.G. (2012). Road Transport of Cattle, Swine and Poultry in North America and Its Impact on Animal Welfare, Carcass and Meat Quality: A Review. Meat. Sci..

[17] Hussnain F., Mahmud A., Mehmood S., Jaspal M.H. (2020). Effect of Transportation Distance and Crating Density on Preslaughter Losses and Blood Biochemical Profile in Broilers during Hot and Humid Weather. Turk. J. Vet. Anim. Sci..

[18] Baker C.J. (1994). Aerodynamics of Poultry Transporters: Implications for Environmental Control. Worlds Poult. Sci. J..

[19] Randall J.M., Streader W.V., Meehan A.M. (1994). Vibration on Poultry Transporters. Worlds Poult. Sci. J..

[20] Vinco L.J., Archetti I.L., Giacomelli S., Lombardi G. (2016). Influence of Crate Height on the Welfare of Broilers during Transport. J. Vet. Behav..

[21] Pinheiro D.G., Machado N.A.F., Barbosa Filho J.A.D., Da Silva I.J. (2021). Computational Analysis of Load Ventilation in Broiler Transport. Eng. Agrícola.

[22] Saeed M., Abbas G., Alagawany M., Kamboh A.A., Abd El-Hack M.E., Khafaga A.F., Chao S. (2019). Heat Stress Management in Poultry Farms: A Comprehensive Overview. J. Therm. Biol..

[23] Spurio R.S., Soares A.L., Carvalho R.H., Silveira Junior V., Grespan M., Oba A., Shimokomaki M. (2016). Improving Transport Container Design to Reduce Broiler Chicken PSE (Pale, Soft, Exudative) Meat in Brazil. Anim. Sci. J..

[24] Machado N.A.F., Barbosa-Filho J.A.D., Ramalho G.L.B., Pandorfi H., Silva I.J.O. (2021). Trailer Heat Zones and Their Relation to Heat Stress Ing Pig Transport. Eng. Agrícola.

[25] Pinheiro D.G., Barbosa Filho J.A.D., Machado N.A.F. (2022). Impact of Load Layout on Internal Ventilation During the Transport of Broilers. Eng. Agrícola.

[26] Gilkeson C.A., Thompson H.M., Wilson M.C.T., Gaskell P.H. (2016). Quantifying Passive Ventilation within Small Livestock Trailers Using Computational Fluid Dynamics. Comput. Electron. Agric..

[27] Li H., Rong L., Zhang G. (2016). Study on Convective Heat Transfer from Pig Models by CFD in a Virtual Wind Tunnel. Comput. Electron. Agric..

[28] He X., Wang J., Guo S., Zhang J., Wei B., Sun J., Shu S. (2018). Ventilation Optimization of Solar Greenhouse with Removable Back Walls Based on CFD. Comput. Electron. Agric..

[29] Ilangovan A., Curto J., Gaspar P.D., Silva P.D., Alves N. (2021). CFD Modelling of the Thermal Performance of Fruit Packaging Boxes—Influence of Vent-Holes Design. Energies.

[30] Ahmadi Babadi K., Khorasanizadeh H., Aghaei A. (2022). CFD Modeling of Air Flow, Humidity, CO2 and NH3 Distributions in a Caged Laying Hen House with Tunnel Ventilation System. Comput. Electron. Agric..

[31] Conselho Nacional de Trânsito (2022). Resolução CONTRAN N^o^ 961, de 17 de Maio de 2022.

[32] Farouk M. (2020). Comparative Study of Hexagon & Square Windcatchers Using CFD Simulations. J. Build. Eng..

[33] Berman A., Horovitz T., Kaim M., Gacitua H. (2016). A Comparison of THI Indices Leads to a Sensible Heat-Based Heat Stress Index for Shaded Cattle That Aligns Temperature and Humidity Stress. Int. J. Biometeorol..

[34] Rodrigues V.C., da Silva I.J.O., Vieira F.M.C., Nascimento S.T. (2011). A Correct Enthalpy Relationship as Thermal Comfort Index for Livestock. Int. J. Biometeorol..

[35] Rodrigues M., Garcia Neto M., Perri S., Sandre D., Faria M., Oliveira P., Pinto M., Cassiano R. (2019). Techniques to Minimize the Effects of Acute Heat Stress or Chronic in Broilers. Braz. J. Poult. Sci..

[36] Machado N.A.F., Martin J.E., Barbosa-Filho J.A.D., Dias C.T.S., Pinheiro D.G., Oliveira K.P.L., Souza-Junior J.B.F. (2021). Identification of Trailer Heat Zones and Associated Heat Stress in Weaner Pigs Transported by Road in Tropical Climates. J. Therm. Biol..

[37] Emesu P., Chepete J.H., Thipe E.L. (2025). Computational Fluid Dynamics Assessment of Natural Ventilation in Three Types of Large-Scale Broiler Poultry Houses in Botswana. Sci. Afr..

[38] Ma H., Tu Y., Yang X., Yang Z., Liang C. (2022). Influence of Tunnel Ventilation on the Indoor Thermal Environment of a Poultry Building in Winter. Build Environ..

[39] Mitchell M.A., Kettlewell P.J. (1998). Physiological Stress and Welfare of Broiler Chickens in Transit: Solutions Not Problems!. Poult. Sci..

[40] Tabase R.K., Van Linden V., Bagci O., De Paepe M., Aarnink A.J.A., Demeyer P. (2020). CFD Simulation of Airflows and Ammonia Emissions in a Pig Compartment with Underfloor Air Distribution System: Model Validation at Different Ventilation Rates. Comput. Electron. Agric..

[41] Machado N.A.F., Barbosa Filho J.A.D., de Sousa A.C., de Sousa A.M., Corrêa W.C., Rodrigues A.A., Cunha B.B. (2024). Numerical Evaluation of Aerodynamic Devices in Mitigating Heat Stress in Pigs During Transport. Eng. Agric..

[42] Seedorf J., Schmidt R.-G. (2017). The Simulated Air Flow Pattern around a Moving Animal Transport Vehicle as the Basis for a Prospective Biosecurity Risk Assessment. Heliyon.

[43] Norton T., Kettlewell P., Mitchell M. (2013). A Computational Analysis of a Fully-Stocked Dual-Mode Ventilated Livestock Vehicle during Ferry Transportation. Comput. Electron. Agric..

